# Whole-Genome DNA Methylation Analysis in Age-Related Hearing Loss

**DOI:** 10.3390/genes16050526

**Published:** 2025-04-29

**Authors:** Marie Valerie Roche, Denise Yan, Yan Guo, Naser Hamad, Juan I. Young, Susan H. Blanton, Feng Gong, Xue Zhong Liu

**Affiliations:** 1 Department of Otolaryngology, University of Miami Miller School of Medicine, Miami, FL 33136, USA; mvr59@miami.edu (M.V.R.); dyan@med.miami.edu (D.Y.); naserhamad.nh48@gmail.com (N.H.); sblanton@med.miami.edu (S.H.B.); 2 Department of Biochemistry and Molecular Biology, University of Miami Miller School of Medicine, Miami, FL 33136, USA; fgong@med.miami.edu; 3 Department of Public Health and Sciences, University of Miami Miller School of Medicine, Miami, FL 33136, USA; yxg835@med.miami.edu; 4 John P. Hussman Institute for Human Genomics, John T. Macdonald Foundation, Department of Human Genetics, University of Miami Miller School of Medicine, Miami, FL 33136, USA; jyoung3@miami.edu

**Keywords:** epigenetics, presbycusis, inner ear, CpG site methylation, hearing loss, DNA methylation, methylation-specific PCR

## Abstract

Background: Presbycusis, also known as age-related hearing loss (ARHL), is the most frequent sensory disability affecting elderly adults worldwide. ARHL is characterized by bilateral, progressive, sensorineural hearing loss that is more pronounced at a high frequency. Conventional factors associated with ARHL include diabetes, hypertension, and a family history of hearing loss. The severity of hearing impairment varies between individuals. The defined causative molecular pathogenesis for ARHL is unknown, thus the identification of underlying pathogenic mechanisms involved in ARHL is imperative for the development of effective therapeutic approaches. Epigenetics is the study of phenotypic changes caused by the modification of gene expression rather than the alteration of a DNA sequence. While it is hypothesized that ARHL could result from undiscovered epigenetic susceptibility, there is a shortage of information on the role that epigenetic modification plays in ARHL. Here we present an investigation on the involvement of DNA methylation in ARHL. Results: Clinical, audiometric and DNA testing, and high-throughput methylation pattern screening were undertaken for ARHL patients and matched control subjects. Our results demonstrate a strong correlation between patients’ hearing measurements and methylation at CpG sites cg1140494 (ESPN) and cg27224823 (TNFRSF25). We identified 136 differentially methylated CpGs that were shared between a high and low audiometric frequency in the patient’s cohort. CpG cites in hearing loss candidate genes, *KCNQ1*, *TMEM43*, *GSTM1*, *TCF25*, and *GSR*, were found to be highly methylated in presbycusis patients as compared to the controls. A methylation polymerase chain reaction (PCR) assay was used to confirm methylation levels at a specific gene locus in ARHL patients and controls. Conclusions: Altered DNA methylation and its impact on gene expression has been implicated in many biological processes. By interrogating the methylation status across the genome of both hearing loss patients and those with normal hearing, our study can help to establish an association between the audiometric patterns and methylation status in ARHL, yielding new avenues for the identification of potential candidate genes for hearing loss.

## 1. Introduction

Age-related hearing loss (ARHL) or presbycusis is the most common sensory disorder present in the elderly population [1]. ARHL arises from the decline in hearing ability that happens with age. Approximately one in three adults over the age of 65 and half of 85-year-olds have hearing loss, showing a consistent decline in hearing [2]. ARHL can also have psychological effects, by contributing to depression, isolation, and dementia [3]. The main characteristic of ARHL is the inability to understand high-frequency components of speech, silent consonants, and a decreased ability in hearing speech in loud environments. A diagnosis of ARHL is established with audiometry, while technologies such as hearing aids and cochlear implantations can alleviate the various symptoms, yet they do not restore normal hearing [4]. This is due in part to the lack of knowledge regarding the underlying causes of this sensory impairment.

Anatomically, ARHL is linked to several auditory structures and divided into several subtypes. Sensory ARHL is caused by the degeneration of the mechanotransducing cochlear inner and outer hair cells. Metabolic ARHL, also known as strial ARHL, is due to reduced function within the stria vascularis. Neutral ARHL results from a deterioration of the auditory nerve [5,6]. Mixed and Intermediate types of ARHL were added in 1993. Many believe that the majority of individuals will exhibit a mixed pathology of ARHL where changes to the peripheral lesions of the inner ear and the central auditory pathways simultaneously could contribute to the progression of ARHL [7]. ARHL is considered a multifactorial disorder, and it involves intrinsic and extrinsic factors. Many factors have been implicated in the development of ARHL: including biological age, sex, the environment, genetics, oxidative stress, and mitochondrial activity, all of which play a role in the aging process, cell death apoptosis, and necrosis [8]. Additionally, risk factors such as medication-induced ototoxicity, hypertension, diabetes, otitis media infection history, a family history of hereditary hearing loss, and the accumulation of noise exposure have also been linked to ARHL [9]. Currently our understanding of this complex disease is limited, and further studies are imperative to satisfy the urgent demand for therapeutic interventions to combat age-related auditory decline.

The epigenome is dynamic and the changes can be caused by environmental conditions and age [10]. The epigenetic regulation of gene expression in the ageing ear coupled with environmental exposure might account for age-related changes to the hearing ability in ARHL patients. Current studies have shown that epigenetics modifications influence the inner ear [11]. A better understanding of epigenetic mechanisms in hearing impairment can support the development of new treatments for hearing loss. The link between epigenetic mechanisms and hearing loss is well established; however, the specific biological mechanism is not well understood [12]. DNA methylation is the most characterized epigenetic modification that affects inner ear functions in ageing [13]. There have been several associations identified between DNA methylation and ARHL. One study has shown that a decrease in the expression of Connexin 26 (Cx26), the protein encoded by the *GJB2* gene which forms gap junctions (GJs), may contribute to the development of ARHL. An increase in CpG methylation at the promoter region of the *GJB2* gene has also been found and this hypermethylation of the promoter region has been associated with Cx26 downregulation [14]. CDH23, also known as otocadherin, a member of a super-family of a calcium-dependent cell-surface adhesion protein, is a component of lateral and stereo ciliary tip links of the inner ear sensory hair, which then controls the auditory process. It has recently been demonstrated that higher CpG site methylation levels in *CDH23* are likely to be associated with ARHL [15].

In this study, we illustrate an approach that can help mitigate the difficult task of identifying the molecular basis of ARHL. We hypothesize that ARHL cases could be derived from alterations in epigenetic regulations. This would explain why the main causes of this disease cannot be found by simply looking at the DNA sequence. The objective of this study is to identify an association between DNA methylation changes and ARHL.

## 2. Methods

### 2.1. Subjects

This study was approved by the University of Miami’s institutional review board. Subjects were recruited from adults attending the outpatient clinic of the University of Miami Ear Institute. This study was performed in accordance with relevant guidelines and regulations. Written informed consent was obtained from all subjects prior to the clinical evaluation and blood sample collection. All patients completed a questionnaire on demographic information including ethnicity and a medical history focusing on the identification of factors with known effects on hearing such as excess noise, ear diseases, ear trauma, radiation exposure of surgery, chronic illness (i.e., diabetes and cardiovascular disease), manifestations of syndromic deafness (i.e., blindness from retinitis pigmentosa, tegumentary, and craniofacial anomalies), and a family history of hearing loss. Simplex and multiplex families were recruited. The inclusion criteria for subjects with ARHL consisted of age greater than 50 years with bone conduction pure tone average ([PTA]) of frequencies 500, 1000, 2000, and 4000 Hz greater than 30 dB HL hearing loss. Exclusion criteria included (1) previous history of exposure to excess environmental and occupational noise; (2) exposure to toxins or drugs with known ototoxic effects; (3) history of temporal bone trauma; (4) history of otologic disorders such as Meniere’s disease, autoimmune hearing loss; (5) average conductive hearing loss greater than 15 dB hearing loss (HL) in one or two ears, measured at 500, 100, and 200 Hz; (6) unilateral or significantly symmetric (greater than 25 dB difference in interaural PTA) hearing loss [16,17].

### 2.2. Audioprofiles

The recruited subjects with ARHL underwent audiological examination according to current clinical standards (Angeli, Bared et al. 2012) [16]. The audiological examination consisted of measurements of air and bone conduction pure-tone thresholds at 500, 1000, 2000, 4000, and 8000 Hertz. Air-conduction threshold curves were classified into one of five audiometric configurations or audioprofiles based on previously reported guidelines [18,19].

1. High-frequency gently sloping: 15–29 dB difference between the average of 0.5–1 kHz and the average of 4–8 kHz.

2. High-frequency steeply sloping: the difference between the average of 0.5–1 kHz and the average of 4–8 kHz is 30 dB or greater.

3. Low-frequency ascending: greater than 15 dB difference between 0.5 and 2 kHz.

4. Mid-Frequency “U” shaped: greater than 15 dB difference between the average of mid-frequency pure tones (1–2 kHz) and that of the low tones (0.25 to −0.5 kHz) and high tones (4–8 kHz).

5. Flat: less than 15 dB difference between the average of 0.25–0.5 kHz, the average of 1–2 kHz, and the average of 4–8 kHz.

The audiometric patterns that were more frequent in the cohort study were “High frequency Steeply Slopping” or HFSS (33%)”, High frequency Gently Slopping”, or HFGS (31%) and “FLAT” (27%), while the other patterns were less prevalent. No statistical significance was found in terms of gender, age, ear side, and PTA values among the audiometric types [16,17]

### 2.3. DNA Isolation and Bisulfite Conversion

Blood samples were collected and genomic DNA was extracted from blood using a standard extraction method. DNA concentrations were measured using the Qubit^®^ dsDNA HS Assay (Invitrogen Carlsbad, CA, USA). For methylation analysis, 500 ng of Isolated DNA was treated with bisulfite using the Epigentek Bisulflash DNA Modification Kit.

### 2.4. Candidate Genes Approach

A list of genes involved in hearing loss was assembled from an intensive review of the literature of sensorineural hearing loss (SNHL) and ARHL, both nonsyndromic and syndromic hearing loss, as well as association and animal models studies.

### 2.5. Array-Based Methylation Assay

The Illumina MethylationEPIC BeadChip was used as array-based methylation profiling. The MethylationEPIC BeadChip (Illumina, San Diego, CA, USA) measures over 850,000 CpG loci across diverse set of functionally relevant genomic regions that include promoters, CpG islands (CGI), CGI shores, intergenic CpGs, as well as gene body. Approximately, 500 ng of genomic DNA were bisulfite-modified using the EZ-96 DNA Methylation Kit (Zymo Research, Irvine, CA, USA) then processed as indicated by Illumina. The BeadChip images were scanned and the data were analyzed using the R software (version 3.2.4). For quality control, probes that had a detection *p*-value < 0.01 were selected for all samples. The Illumina assay utilizes a pair of probes (a methylated probe and an unmethylated probe) to measure the intensities of the methylated and unmethylated alleles at the interrogated CpG sites. The methylated and unmethylated signal intensities were quantile-normalized for each individual probe. Subsequently, both β and M values were estimated. β values represent the ratio of the methylated signal intensity to the sum of both unmethylated and methylated signals after background subtraction (β values range from 0 to 1, 0 corresponding to a completely unmethylated site, while 1, respectively, represents a fully methylated site). M values were obtained; they have been shown to have superior statistical properties such as homoscedasticity: M values are logit transformations of the β values, which are *M* = log (β/1 − β). *M*-values near 0 signify a similar intensity between the methylated probe and the unmethylated probes, corresponding to a CpG site that is half methylated; while positive *M*-values suggest that more molecules are more methylated than unmethylated, negative *M*-values indicate the contrary.

### 2.6. Data Analysis

The 850K methylation array data underwent processing, quality control, and normalization procedures using Illumina GenomeStudio software (version 2.0.5). Samples that failed quality control were removed from subsequent analysis. The relationship between hearing loss and CpG methylation levels was assessed through regression analysis using glm package in R. Hearing loss status served as the outcome variable, and each CpG site’s methylation level served as the primary predictor adjusting for age, sex, and race. Hearing loss was represented both as a binary (logistic regression) and continuous (linear regression) variable across hearing tests at different frequencies. Benjamini Hochberg’s multiple-test-corrected *p* < 0.05 was used as the significant threshold [20]. CpG sites were annotated to genes based on the Genecode v44 GTF, with annotations extending by 1500 bp both upstream and downstream of the genes. Pathway analysis was conducted utilizing WebGestalt [21]. Gene set enrichment analysis was performed using GSEA (version 4.3.3). Principal component analysis (PCA) was performed using Python (version 3.12). Heatmap and unsupervised clustering analysis was carried out using the heatmap package in R [22,23]. Manhatton plots were drawn by the ggplot2 package in R.

### 2.7. Methylation-Specific Polymerase Chain Reaction Validation

Methylation-specific PCR was performed to validate the methylation data at selected loci. Primers sets were designed using MethPrimer to discriminate between methylated CpG sites from unmethylated alleles. The DNA samples were modified by bisulfite conversion using the Bisulflash DNA Modification Kit (Epigentek, New York, NY, USA) according to the manufacturer’s protocol. Optimized PCR reactions were conducted using the GoTaq Green Master Mix (Promega, Madison, WI, USA). The PCR conditions were as follows: reaction volume, 25 μL; primers, 10 pM; template genomic DNA, 100ng; denaturation at 95 °C for 5 min and at 95 °C for 30 s; and at 60 °C for 30 s and 72 °C for 30 s for 35 cycles followed by a 5 min final extension at 72 °C. Primer sequences for the CpG sites cg11404945 (*ESPN*) and cg27224823 (*TNFRSF25*) tested are shown in Table 1.

## 3. Results

### 3.1. Cohort Description

The demographic description of the full cohort, including the audiometric results, can be found in Table 2. The average age in the ARHL group is 65.42, which is comparable to 60.67 in the control group. The median ages are 66 for the ARHL group and 61 for the control group. In terms of sex distribution, the ARHL group consists of 45% female and 55% male, while the control group has a higher female proportion at 67%, with 33% male. The audiometric results confirm the hearing loss condition and normal condition for the ARHL and control groups, respectively.

### 3.2. Robust Association Between ESPN and TNFRSF25 CpG Sites with Patients’ Hearing Thresholds

We investigated a subset group consisting of 14 ARHL patients who met the audiometric inclusion criteria, as previously described. The average age of the subjects was 63 ± 10 years. All subjects were prescreened and found negative for mutations in the *GJB2* gene and *GJB6* deletions (GJB6-D13S1830 and GJB6-D13S1854) as well as pathogenic mitochondrial variants (MT-RNR1:111.1555 A>G; MT-TL1: m.3243 A>G). The Illumina MethylationEpic BeadChip microarray was used to perform an epigenome-wide association study.

**Figure 1:**
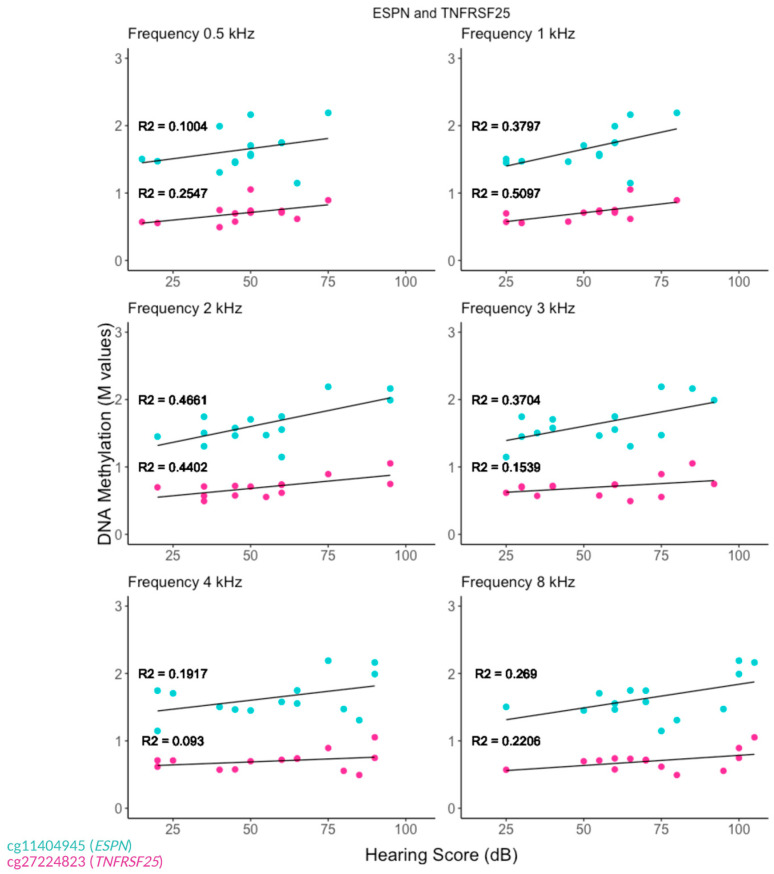
Two adjacent CpG sites, cg11404945 and cg27224823, located between ESPN and TNFRSF25, show an increase in methylation levels as the patients’ hearing deteriorates. A positive correlation between the hearing level and the level of CpG site methylation at each frequency is detected in the linear regression analysis. The CpG sites cg11404945 (ESPN) and cg27224823 (TNFRSF25) are located at the genomic positions 6521138 and 6521268 on chromosome 1, respectively. Mutation in ESPN can cause DFNB36, nonsyndromic-dominant hearing loss, or USH1M. TNFRSF25 plays a vital role in regulating cell proliferation, differentiation, and apoptosis.

**Figure 2:**
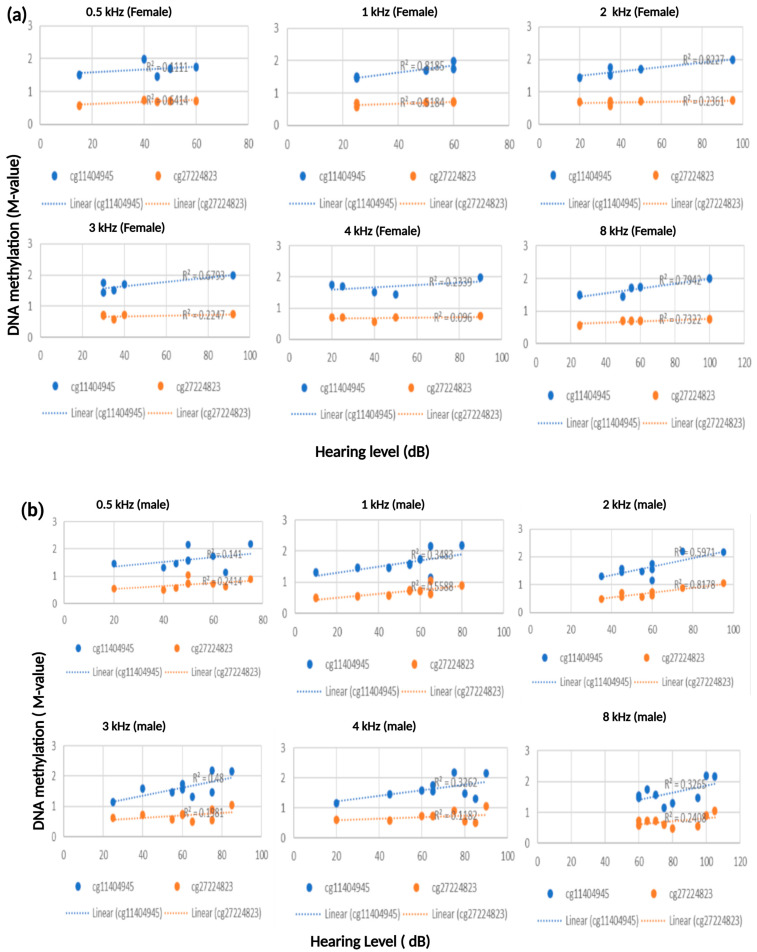
(**a**) In a sex-stratified manner, in all frequencies, a significant correlation between the hearing measurements and the level of methylation in the *ESPN* and *TNFRSF25* genes has been observed in females, (**b**) as well as in the male patients from our cohort.

Linear regression analysis was used to assess the relationship between the patients’ hearing threshold (outcome) and the *M*-value scores (predictor). We have determined an association between ARHL patients’ hearing thresholds and the *M*-values obtained for the individual CpG sites. A notable finding from our analysis is that two contiguous CpGs (cg114044945 and cg2724823), located downstream of the *ESPN* gene and at the 3′UTR of the tumor necrosis factor receptor gene *TNFRSF25*, have shown a positive correlation with the hearing thresholds (Figure 1). This effect is observed at every audiometric frequency (0.5 kHz–8 kHz) in both females and males (Figure 2a,b). These two adjoining CpG cites increase methylation as patients hearing aggravates. These CpGs are located in an area identified as a distal enhancer by the ENCODE Registry of Candidate cis-Regulatory Elements (cCREs). Furthermore, they are positioned in a CpG island and are in close proximity to expression quantitative trait loci (eQTL) for both *ESPN* and *TNFRSF25* genes, as identified in GTEx (Supplementary Figure S1).

### 3.3. Correlation Between Patients’ DNA Methylation Level and Hearing Score at 8 kHZ

**Figure 3:**
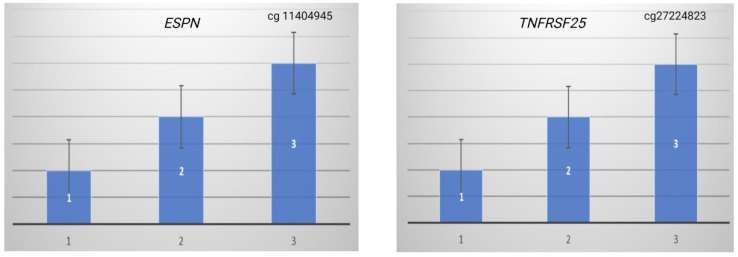
At the high audiometric frequency (8 kHz), patients with severe hearing loss (group 3) showed higher methylation level in the CpG sites cg11404945 (*ESPN*) and cg27224823 (*TNFRSF25*) compared to the methylation level in the patients with moderate hearing loss (group 2) and subjects with normal hearing or mild hearing (group 1), with a standard deviation of 0.5 (*n* = 3) per group.

ARHL is known to be typified at high frequencies. In this study, we proceeded to divide our subjects’ patients based on hearing audioprofiles at 8 kHz. Group one comprised patients with mild hearing loss (26 dB–40 dB), the second group was made up of patients with moderate hearing loss (41 dB–70 dB), while group three included patients with severe hearing loss (90+ dB) (Figure 3). Our data show a higher level of methylation in the ESPN and TNFRSF25 CpG sites in patients with severe hearing loss at 8 KZ compared to the patients with mild and moderate hearing loss.

### 3.4. Methylation-Specific PCR Validation

**Figure 4:**
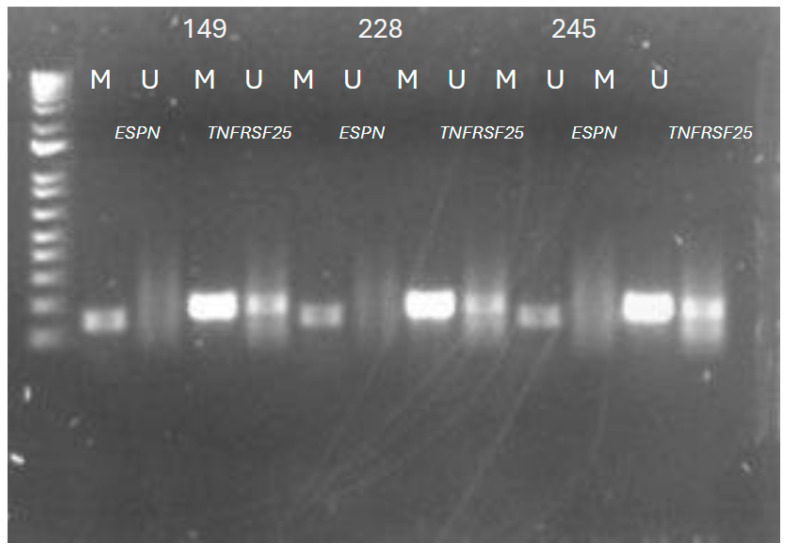
Validation of the methylation of CpG sites cg11404945 (*ESPN*) and cg27224823 (*TNFRSF25*) confirm the results obtained from the Illumina microarray. The Presbycusis patients’ DNA samples (149, 228, 245) were amplified after bisulfite treatment. M refers to methylated, U refers to unmethylated.

The validation of selective methylated gene-specific CpGs identified from the Illumina MethylationEPIC BeadChip was performed using Methylation-Specific PCR of the ARHL patients’ genomic DNA. We showed that the methylation status of the identified CpG cites were consistent with the Methylation Epic microarray (Illumina) assay, which interrogates the DNA methylation of 865,000 CpG sites. (Figure 4). Therefore, the methylation status of the *ESPN* and *TNFRSF25* genes suggest the possible utilization of these hearing loss subjective genes as epigenetic signatures, hence showing that DNA methylation can be used as a tool for epigenetic-based therapeutic strategies.

### 3.5. Identification of Differentially Methylated CpG and Genes

Linear regression analyses were carried out at each audiometric frequency using the audiometric results as the outcome and the *M*-value as the predictor, while adjusting for age and sex.

**Figure 5:**
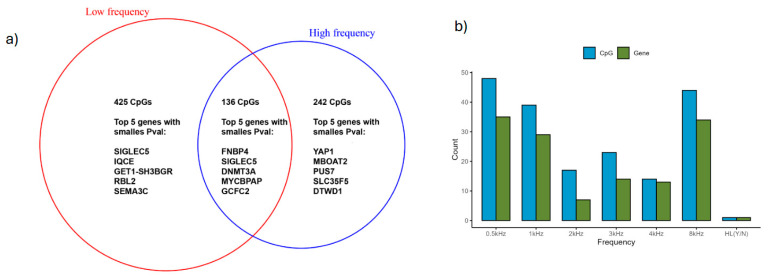
(**a**) Venn diagram displaying CpGs in low- and high-audiometric-frequencies analysis. A total of 136 CpGs were common in the two groups. CpG sites with *p* < 0.05 are shown. (**b**) Number of CpG sites and genes found at each audiometric frequency.

Differentially methylated CpG sites were identified between presbycusis patients and controls using the following selection criteria: (1) linear regression *p* < 0.05 in all frequency analyses and (2) a log2 fold change greater than 0, indicating higher methylation levels in the hearing loss group compared to the control group. The low-frequency group (0.5 kHz, 1 kHz, and 2 kHz) had 425 differentially methylated CpGs; the high frequency group (3 kHz, 4 kHz, and 8 kHz) had 242 differentially methylated CpGs. Additionally, 136 differentially expressed CpGs were shared by the high- and low-frequency groups (Figure 5).

**Figure 6:**
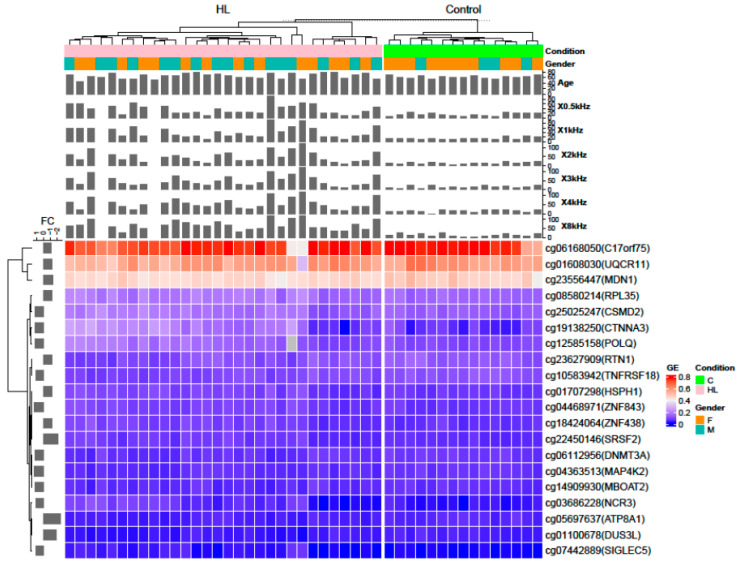
Heatmap analysis of the top 20 highly differentially methylated CpG sites using linear regression analyses with low (0.5 kHz, 1 kHz, and 2 kHz) and high (3 kHz, 4 kHz, and 8 kHz) audiometric frequencies of presbycusis patients and controls. The selection criteria are as follows: (1) linear regression *p* < 0.05 in all frequency analyses; (2) CpG must be in the promoter region of the genes (3) log2 fold change greater than 0, indicating higher methylation levels in the hearing loss group compared to the control group samples. The bar plot on the left side denotes the fold change of the corresponding CpG sites between hearing loss group and control group.

**Figure 7:**
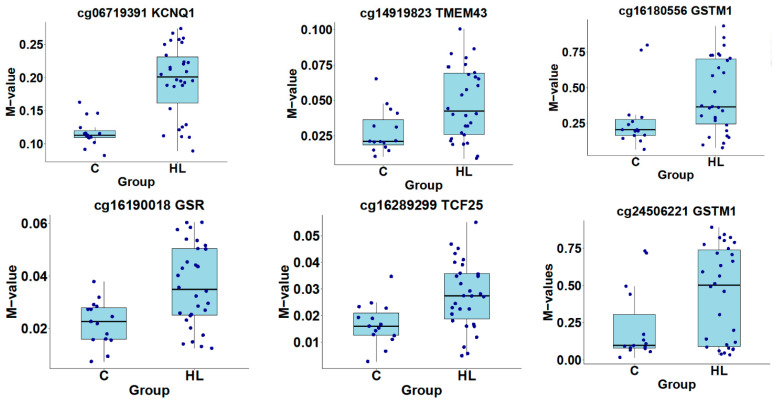
Turkey box plots showing distribution of β values of the top 6 CpG sites from the hearing candidate genes where the methylation level is higher in the presbycusis subject group versus the control group. The box plot displays the data within the interquartile range and the median is represented by a solid black line.

**Figure 8:**
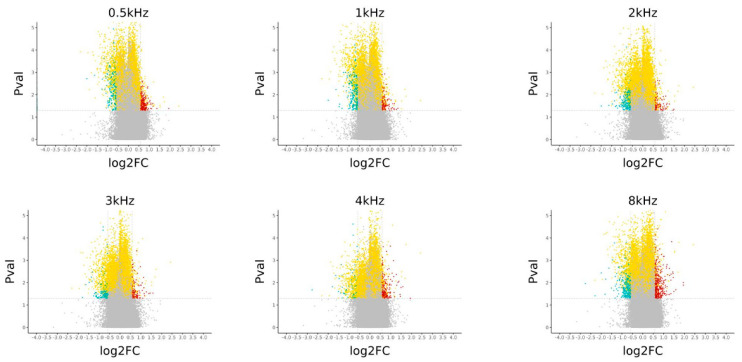
Volcano plot of −log10 adjusted *p*-value against log2 fold change, representing the methylation difference among CpG sites identified between the control group and the presbycusis group at low audiometric frequencies (0.5 kHz, 1 kHz, and 2 kHz) and at high audiometric frequencies (3 kHz, 4 kHz, and 8 kHz).

A cluster heatmap shows the top 20 highly differentially methylated CpGs using linear regression for low and high audiometric frequencies. The selection criteria are the same as in the previous Venn diagram, with an additional criterion that the CpG must be in the promoter region of a gene (Figure 6). Turkey box plots show the distribution of β values for the top six CpG sites from the hearing candidate genes and the identified CpG sites are in the *KCNQ1*, *TMEM43*, *GSTM1*, *TCF25*, and *GSR* genes (Figure 7). Volcano plots were constructed to show the methylation difference between CpG sites identified between the control group and the hearing loss group (Figure 8).

**Figure 9:**
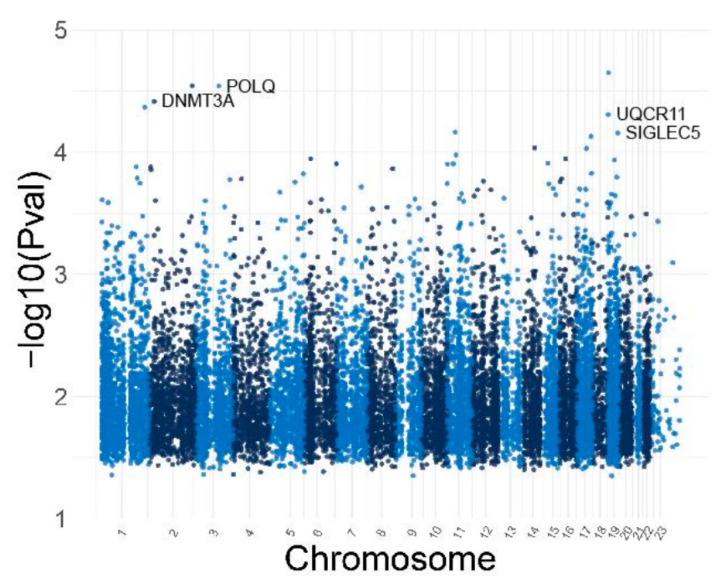
Manhattan Plot depicting the −log 10 *p*-value of the association between CpG sites and hearing threshold at low audiometric frequencies (0.5 kHz, 1 kHz, and 2 kHz) and high audiometric frequencies (3 kHz, 4 kHz, and 8 kHz). The genes with significantly associated CpG sites are shown at the top. The *p*-values are represented in genomic order by chromosomes and positions on the chromosome (x-axis).

**Figure 10:**
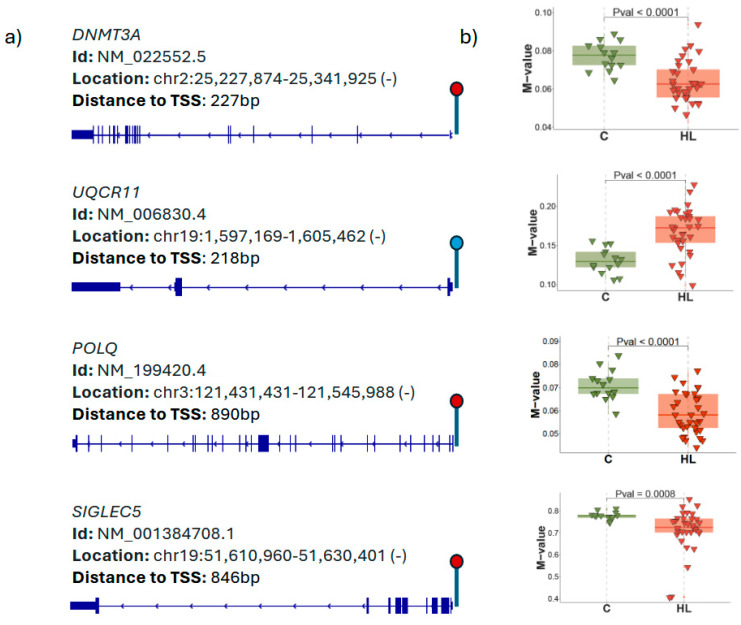
(**a**) The location and ID of the identified CpG sites and their distance to the transcription start sites of the corresponding genes are shown. The red and blue circle depicts the transcription start sites. (**b**) Box plots indicating the distribution of methylation β value for CpG sites with *p* < 0.05 overlapping between high and low audiometric frequency in the case-control analysis.

Manhattan plots demonstrating the association between the hearing scores and DNA methylation of ARHL patients for low and high frequencies are shown. The *p*-values are depicted in genomic order by chromosomes and positions on the chromosome (x-axis). All CpGs labeled in the Manhattan plots show nominal association (*p* < 1 × 10^−5^) with the ARHL patients’ hearing threshold at each audiometric frequency (0.5 kHz–8 kHz). The list of nominally significant CpGs (*p* < 1 × 10^−5^) sorted by their p-value for low and high audiometric frequencies are depicted. The CpG sites that reached genome-wide significance are located in the promoter regions of *DNMT3A*, *POLQ*, *UQCR1*, and *SIGLEC5* (Figure 9 and Figure 10).

## 4. Discussion

In this study, we performed an epigenome-wide association method, and we have identified CpG sites associated with ARHL. The methylation of DNA CpG sites is linked with disease state, ageing, and environmental factors. Given the polygenic and multifactorial nature of ARHL, with this study, we inquired into the extent of the involvement of epigenetic alterations in this disorder.

We present a strong correlation between an elevated CpG methylation level in two contiguous sites located in the intergenic region between the genes *ESPN* and *TNFRSF25*. These CpG sites localize in a region predicted to act as a transcriptional enhancer and that contains eQTLs for *ESPN* and *TNFRSF25*. This suggests that the altered methylation at these sites could affect the expression of the *ESPN* and *TNFRSF25* genes. Espins are actin bundling proteins found in hair cells, stereocilia. The mutation of the *Espn* gene can cause hearing loss and vestibular dysfunction in the *jerker* mouse [24]. Additionally, an in-frame deletion of human *ESPN* has been associated with USH1M [25]. The TNFRSF25 (TNF Receptor Superfamily Number 25) with an ability to bind necrosis factors via an extracellular cysteine-rich domain and has been shown to play a role in the death receptor signaling pathway and apoptosis [26]. Using a sex-stratified strategy, we have also determined at all frequencies that there is a positive correlation between our patients’ hearing measurement and the level of methylation in the *ESPN* and *TNFRSF25* as well. Future comprehensive studies are warranted to test the concord of the *ESPN* and *TNFRSF25* methylation and gene transcription levels. Our results demonstrate that the epigenetic field, complimentary to our current knowledge of the gene networks, can enhance our understanding of the genome in response to intracellular and environmental factors.

The discovery that methylation at the promoter regions of *DNMT3A*, *UQCR11*, *POLQ*, and *SIGLEC5* has a protective effect against hearing loss suggests a multifaceted mechanism by which epigenetic regulation preserves auditory function. Each of these genes plays distinct roles in cellular processes, and their repression through promoter methylation could collectively mitigate factors that contribute to hearing impairment. *DNMT3A* is responsible for *de novo* DNA methylation, which often leads to the silencing of genes [27]. While this function is essential for normal development, excessive DNMT3A activity could result in the inappropriate methylation and silencing of genes necessary for cochlear function [28]. For instance, *DNMT3A* could target genes that maintain the health of cochlear hair cells, the sensory cells responsible for detecting sound [29]. If these hair cells are deprived of critical gene activity, they become more susceptible to damage from environmental factors like noise exposure or aging. Methylation at the *DNMT3A* promoter would reduce its expression, thereby preventing the over-methylation of other important genes. This balanced reduction in *DNMT3A* activity could help preserve the expression of genes essential for maintaining the structural and functional integrity of the cochlea, protecting against the cellular stress and damage that lead to hearing loss. This promoter sequence analysis also reveals that the methylation level of one CpG site in the *UQCR11* gene was significantly higher in patients compared to the controls. Interestingly, located in mitochondria, mouse *Uqcr11* has been shown to reduce apoptosis and alleviate oxidative stress both in vitro and in vivo [30] The human *UQCR11* (Ubiquinol–cytochrome c reductase) gene is mapped to chromosome 19p13.3. It encodes a protein of 56 amino acids. It is a component of the ubiquinol–cytochrome c oxidoreductase, a multi-subunit transmembrane complex that is part of the mitochondrial electron transport chain which drives oxidative phosphorylation.

An expression analysis revealed that Uqcr11 is expressed in cochlear and vestibular hair cells as well as spiral ganglion neurons in the inner ear (SHIELD; https://shield.hms.harvard.edu). As the methylation level in one CpG site located in the promoter region of UQCR11 is increased in ARHL patients compared to the controls, this indicates that the DNA methylation of UQCR11 as an epigenetic modification might play an important role in the occurrence of ARHL by altering the anti-apoptosis/oxidative stress properties of UQCR11.

POLQ (DNA polymerase theta) plays a key role in DNA repair, particularly in the repair of double-strand breaks via alternative end joining (alt-EJ) [31]. While this pathway is crucial for preventing genomic instability, POLQ is also known for its error-prone nature, which can lead to mutations. In the context of the cochlea, where cells are exposed to high levels of oxidative stress due to their energy demands, uncontrolled DNA repair processes might contribute to mutations or genomic instability, increasing the risk of cellular dysfunction. Methylation at the POLQ promoter could reduce the gene’s expression, thereby limiting the use of this error-prone DNA repair mechanism. By restricting POLQ activity, cochlear cells may rely more on accurate repair pathways, preserving genomic integrity and reducing the accumulation of mutations that could otherwise impair the cellular function and contribute to hearing loss. This protective effect could be particularly important in age-related or stress-induced hearing loss, where DNA damage accumulates over time.

*SIGLEC5* is an immunoregulatory gene involved in modulating the immune response, particularly through the inhibition of pro-inflammatory signaling [32]. Chronic inflammation in the cochlea is a known contributor to hearing loss, as it can lead to tissue damage, the disruption of cellular processes, and oxidative stress [33]. Dysregulated immune activity may exacerbate hearing impairment, especially in cases of noise-induced or infection-related hearing loss. Methylation at the SIGLEC5 promoter could help fine-tune the inflammatory response in the cochlea by reducing excessive immune signaling. By preventing the overexpression of SIGLEC5, this epigenetic modification could protect the cochlea from inflammation-induced damage, promoting a more controlled and protective immune environment. This would help preserve cochlear cells and auditory function over time, reducing the risk of inflammation-related hearing loss. There are several limitations to our study that should be recognized. First, the limited sample size of ARHL patients helped us in achieving an association study; future studies will be conducted by adding age–gender-matched control samples from the same geographical areas as that of the subject. A second limitation is the usage of DNA samples from the ARHL patients’ peripheral blood due to the inaccessibility of the inner ear tissues. In conclusion, our study highlights the implication of DNA methylation, a main epigenetic marker in regulating gene transcriptional regulation and preserving genome stability association with ARHL.

## Figures and Tables

**Table 1:** Primers were designed using MethPrimer. The primer sets used for the amplification were labeled as methylated (M) and unmethylated (U).

Primer Set	Forward Primer	Reverse Primer	Size, bp
*ESPNM* cg11404945	TATGTTTTGATTTATCGGATTTG C	GAAAATATAAACGCAACAACGAA	146
*ESPNU* cg11404945	GTTTTGATTTATTGGATTTAGTG G	ACAAAAATATAAACACAACAACA	145
*TNFRSF25 M* cg27224823	GTCGTTTTCGAGATTAGTAGTAC G	AAAACGCCTAAATAACAAATAAAC	179
*TNFRSF25 U* cg27224823	GGTTGTTTTTGAGATTAGTAGTA TG	CCAAAACACCTAAATAACAAATAAA CAC	182

**Table 2:** Cohort description.

	ARHL (*n* = 31)	Control (*n* = 15)
Age		
Mean (SD)	65.42 (10.03)	60.67 (4.06)
Median [Q1, Q3]	66 (57, 74)	61 (58.5, 62)
Sex		
Female	45%	67%
Male	55%	33%
Audiometric		
0.5 kHz Mean (SD)	39.03 (20.85)	15 (6.58)
1 kHz Mean (SD)	42.26 (23.55)	15 (4.83)
2 kHz Mean (SD)	50.16 (26.81)	15.33 (5.62)
3 kHz Mean (SD)	50.71 (26.33)	15.33 (6.94)
4 kHz Mean (SD)	55.97 (27.46)	13.33 (6.24)
8 kHz Mean (SD)	69.19 (24.79)	14.67 (6.18)

## Data Availability

The datasets generated and/or analyzed during the present study are available from the corresponding author on reasonable requests.

## Supplementary Materials

The following supporting information can be downloaded at: https://www.mdpi.com/article/10.3390/genes16050526/s1, Figure S1: UCSC genome browser tracks. Top track (custom) showing the location of the CpGs that are associated with hearing score (cg114044945 and cg2724823, orange bars) and the location of the eQTL variants as identified by GTEx (yellow bars, https://gtexportal.org/). Second track: gencode genes. Third track: ENCODE regulatory elements (cCREs). Bottom track: CpG Islands. The rs2986758 is an eQTL for both ESPN and TNFRSF25, whereas rs 147172610 and 54481403 are eQTLs for ESPN.
