# Supplementary material for: Whole-Genome DNA Methylation Analysis in Age-Related Hearing Loss
Source: Genes (Basel). 2025 Apr 29;16(5):526. doi: 10.3390/genes16050526 (PMC12111640; doi:10.3390/genes16050526)
Supplement: Supplementary file 1 [file genes-16-00526-s001.zip › genes-3582800-supplementary.pdf]

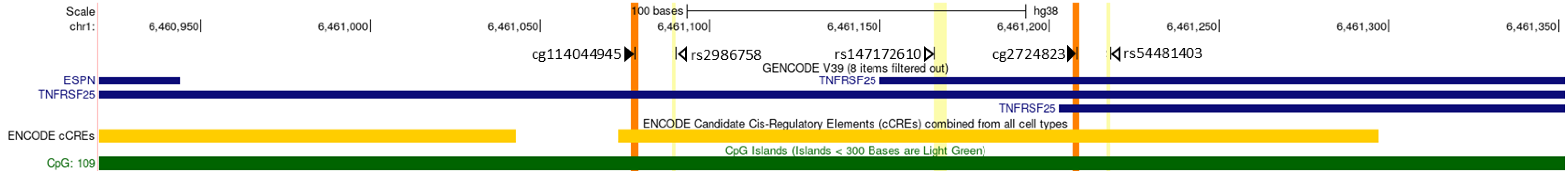


**Supplementary figure S1**: UCSC genome browser tracks. Top track (custom) showing the location of the CpGs that associate with hearing score (cg114044945 and cg2724823, orange bars) and the location of the eQTL variants as identified by GTEx (yellow bars, https://gtexportal.org/). Second track: Gencode genes. Third track: ENCODE regulatory elements (cCREs). Bottom track: CpG Islands. The rs2986758 is an eQTL for both *ESPN* and *TNFRSF25*, whereas rs 147172610 and 54481403 are eQTLs for *ESPN*.

https://genome.ucsc.edu/cgibin/hgTracks?db=hg38&lastVirtModeType=default&lastVirtModeExtraState=&virtModeType=default&virtMode=0&nonVirtPosition=&position=chr1%3A6441956%2D6478124&hgsid=2517521723_nyE6ot4GnwY3STbxMCbVIBcaQOfC
